# Fewer ischemic strokes, despite an ageing population: stroke models from observed incidence in Norway 2010–2015

**DOI:** 10.1186/s12913-019-4538-7

**Published:** 2019-10-16

**Authors:** Kim Rand, Fredrik Andreas Dahl, Joe Viana, Ole Morten Rønning, Kashif Waqar Faiz, Mathias Barra

**Affiliations:** 1 Health Services Research Unit, Akershus University Hospital, Lørenskog, Norway; 2 Institute of Clinical Medicine, Faculty of Medicine, University of Oslo, Oslo, Norway; 3 Centre for Connected Care, Oslo University Hospital, Kirkeveien, 166 Oslo, Norway; 4 Division of Medicine, Akershus University Hospital, Lørenskog, Norway

**Keywords:** Stroke incidence, Epidemiology, Statistical modelling, Cerebrovascular accident, Ischemic stroke, Hemorrhagic stroke

## Abstract

**Background:**

Stroke incidence rates have fallen in high-income countries over the last several decades, but findings regarding the trend over recent years have been mixed. The aim of the study was to describe and model temporal trends in incidence of stroke by age and sex between 2010 and 2015 in Norway, and to generate incidence projections towards year 2040.

**Methods:**

All recorded strokes in Norway between 2010 and 2015 were extracted from the National Patient Registry and the National Cause of Death Registry. We report incidence by age, sex, and year; in raw numbers, per 100,000 person-years, by WHO and European standard populations; and generated statistical models by stroke type, age, sex, and year; and projected stroke incidence toward year 2040.

**Results:**

The data covered 30.1 million person-years at risk, 53431 unique individuals hospitalized with a primary stroke diagnosis, and 6315 additional individuals registered as dead due to stroke. From 2010 to 2015, individuals suffering stroke per 100,000 person-years dropped from 239 to 195 (208 to 177 excluding immediate deaths). The decline was driven by ischemic strokes, with a statistically non-significant time trend for hemorrhagic stroke.

**Conclusions:**

The age-dependent incidence of ischemic strokes in Norway is declining rapidly, and more than compensates for the growth and ageing of the population. Comparisons with historic incidence statistics show that the reduction in incidence rates has accelerated over the last two decades.

## Background

Decision making and resource allocation for future stroke care and prevention depends on up-to-date information regarding trends for incidence and mortality of stroke. Previous systematic reviews have reported a marked decrease in stroke incidence in high-income countries (North America, western and central Europe, Australasia, and high-income Asia Pacific) over the last decades [1–3]. In contrast, some recent studies have indicated increasing incidence [4, 5]. At present, there is a lack of up-to-date assessments of current temporal trends in stroke incidence [6].

Norway, with a population that passed 5 million in 2012, is a comparatively wealthy country with a high life expectancy (81.9 years as of 2015) and a universal public health care system [7]. As observed in other high-income countries [8, 9], incidence rates for strokes have declined in Norway over the last couple of decades, from estimates of approximately 400 per 100,000 person years at risk (PY) before 2000 [10, 11] to around 200 per 100,000 PY based on reports by the National Institute of Public Health (NIPH) in 2015 [12]. However, the NIPH reports may not accurately represent yearly incidence: the reported numbers reflect all hospitalizations overlapping with each particular year, such that a patient admitted in 2014 and discharged in 2015 would be counted in both years. This double counting inflates estimates, both of hospitalizations and of number of unique individuals inflicted. Furthermore, the provided observations are not broken down by age and sex (instead they are limited to standardized incidence rates), and have not been used to generate a prediction model for stroke incidence.

Knowledge regarding trends in incidence and case fatality rates is important to assist in healthcare planning. However, reported trends are only meaningful insofar as they are based on studies with similar definitions, methods, and data presentation. A comprehensive study of changes in incidence in a well-defined population may provide useful results. The aim of the present study was to describe and model recent temporal trends in incidence of stroke and transient ischemic attacks (TIA) by age and sex between 2010 and 2015 in Norway, and to generate projections for stroke incidence between 2015 and 2040.

## Methods

### Diagnoses and validity

We were concerned with cerebrovascular accidents (CVAs) classified as hemorrhagic strokes (intracerebral hemorrhages, ICH, ICD-10 code I61), ischemic strokes (acute cerebral infarction ACI, I63), and stroke of undetermined type (I64), which is used when a stroke is assumed, but confirmation by brain imaging is unavailable. Matching the stroke definition used by the Norwegian Stroke Registry [13], we did not include I60 subarachnoied hemorrhage in our study. Since computer tomography (CT) and/or magnetic resonance imaging (MRI) are essential elements in current stroke diagnosis protocols, the validity of stroke diagnoses in live patients is relatively high [14, 15]. For deaths with no preceding hospitalization, clinical judgement plays a greater role, with comparatively lower validity. With changes in treatment and technology, the I64 diagnosis is now predominantly used when stroke is the assumed cause of death in patients without previous hospitalization, since autopsies and brain imaging are rarely administered in such cases. While stroke as cause of death may have relatively high validity, clinical judgement is not well suited to determine whether said stroke was hemorrhagic or vaso-occlusive in nature. Consequently, we report separately by stroke diagnosis (I61, I63, and I64) for hospitalizations, but limit reporting and modelling of incidence including deaths to the any stroke-category.

In addition to stroke diagnoses, we were interested in patients diagnosed with transient ischemic attacks (TIA; G45). We report on TIA because the delineation between TIA and ischemic stroke may have shifted over time [15], such that cases that might previously have been considered TIAs now are more likely to be categorized as small ischemic infarction following brain imaging with evidence of lesions. Since TIA diagnoses may still be assigned based on clinical expertise unaided by neuroimaging, TIA diagnoses have lower validity than stroke diagnoses. By its definition of being transient - symptoms should resolve within 24 h - TIAs should never be indicated as cause of death.

### Cohort of interest

Individuals suffering from stroke are typically hospitalized, with the important exception of cases in which the patients die before hospitalization. Since changes in health-care and organization could impact the likelihood of reaching or being admitted to a hospital prior to death, estimates of population incidence should ideally include those who die prior to hospitalization. In this study, we combined data from two sources: the Norwegian Patient Registry (NPR), and the Norwegian Cause of Death Registry (NCDR). The cohort of interest was defined as all persons recorded with at least one primary stroke diagnosis or TIA in either NPR or NCDR between 2010 and 2015. Since diagnostic data is limited to assigned diagnostic codes, first-ever strokes are not uniquely identifiable.

## Sources of data

Norwegian Patient Registry: The NPR collates information on all treatment-related activity in Norwegian public hospitals. As there are no private health institutions performing emergency treatment of stroke patients in Norway, NPR covers *all* treated strokes. A standardized subset of fields from all electronic patient journals, plus various administrative data, is automatically transferred from all hospitals to NPR. For all patients in the cohort of interest, we received information regarding all hospital stays between 2010 and 2015. Variables included anonymized id, year of birth, sex, municipality of habitation, date of start and end of each admission, admitting ward, and all assigned ICD10-codes.

Norwegian Cause of Death Registry: For the cohort of interest, we received all recorded deaths, id, date of death, location (municipality), institution (when applicable), municipality of habitation, and all assigned ICD10 codes for cause of death.

Statistics Norway: We extracted data regarding population size and composition, by sex, yearly age, and municipality for 2010–2017, and projections for 2018–2040 [16, 17].

### Merging and periodization of records

Both guidelines and clinical practice dictate that patients with stroke symptoms should be admitted to hospital (i.e. for inpatient stay). Subsequent to initial hospitalization, stroke patients frequently have one or more outpatient visits. Outpatient visits are thus considered uninformative as to stroke incidence, and were excluded from the analysis.

We were interested in determining the number of strokes per year per demographic subgroup (age, sex) in terms of number of individuals afflicted, and in terms of number of treatment episodes. A treatment record was considered as a unique episode if it was separated from previous records with the same diagnosis by at least 24 h. This is the standard approach used by Norwegian authorities when merging hospital stays (see e.g. [12]). Moves between hospitals or hospital wards will be merged, and thus not result in double counting using this rule. While we cannot exclude the existence of cases of double counting for patients if they were to be discharged early and later readmitted, this does not reflect the practice or guidelines, and primary stroke diagnoses should only be recorded for direct admissions. Consequently, stroke records for the same patient on adjacent dates were merged. Merging of records was done separately by CVA sub type, and combined for any stroke (ischemic + hemorrhagic + undetermined). For example, a patient registered with stays at two different hospitals with the end date in the first one day before the start date of the second, with both stays listed as having primary diagnosis I61 would be merged. If the two stays were recorded with I61 in one hospital and I63 in the other, the periods would be counted as separate in analyses of ischemic strokes and hemorrhagic strokes, but as one unit for analyses of any stroke. If the stays were separated by more than 1 day, they would be counted as unique episodes in all conditions. As using the merging rule for any stroke allowed merging of adjacent records with different stroke diagnoses, the sum of reported individuals and reported treatment episodes for ischemic, hemorrhagic, and undetermined is slightly higher than corresponding numbers for any stroke. For the analyses of any stroke, treatment episodes were also merged with recorded deaths for which stroke was listed as the primary cause, in cases where hospitalization and death were separated by less than 24 h.

After the merging of recorded observations into episodes, the treatment episodes were assigned to a unique year by their start date. Thus, an episode lasting from 2011 to 2012 would count only for 2011. When reporting on the number of unique individuals suffering strokes, we report year-unique individuals; The ICD-10 codes reported in patient journals, and collated by NPR, do not specify whether the patient in question has suffered other strokes previously. Consequently, if we were to report on only the first observed stroke per individual, an artificial decline would be created; all observed individuals would be reported for the first observed year (2010), regardless of how many previous strokes they had suffered, while subsequent years would only count individuals for whom no strokes were observed earlier in the observation period. By reporting the number of unique individuals with episodes starting in each year, this artificial decline was avoided.

### Descriptives

We report the observed number of stroke episodes and the number of unique individuals per year per diagnosis, including the aggregate stroke-category any stroke, separately for men and women, based only on hospital data. For any stroke we also report episodes and year-unique individuals counting deaths from stroke with no preceding hospitalization. Numbers are reported as observed, per 100,000 person-years (PY), and for the WHO- and European standard populations [18, 19].

We graphically present the number of strokes by age and sex for each year 2010–2015. We have calculated and graphically present the moving 12-month sum of observed unique patients. With this procedure, the first observation would cover the whole of 2010, the second would cover February 1st 2010 through January 31st 2011, and so on until the final observation would cover the whole of 2015. The choice of 12-month periods were made so as to avoid potential differences caused by seasonal variation; while seasonal variation in actual stroke incidence is expected to be limited [20], observed variations could occur due to structural factors influenced by seasons, such as altered staffing during vacations, or prolonged patient transportation times in the winter.

### Statistical model for incidence

The statistical model was based on the assumption that strokes are unrelated to each other, and can be modelled as a Poisson point process [21]. Since men and women have different risk profiles for strokes, we modelled them separately. Stroke incidence in the very young is likely to have different causes from strokes in adults, and where stroke risk increases rapidly with age in adults, the highest risk for children is shortly after birth. Thus, we modelled children (< 18 years) separately from adults, with children below 1 handled separately from 1 to 17 year olds. Due to the low number of observed strokes in children, the child model was not split by sex. Strokes in children contribute very little to the overall stroke rate in the population, and were included in the modelling effort in this study primarily for completion, and to allow full predictions of stroke rates using standard populations. We tested a small pool of candidate statistical models including combinations of the following predictors: *a* (age); *a*^*2*^; *Y* (year since 2010); Y^2^; *F* (a dummy variable for female sex); an interaction term *a:Y*; and a random intercept for the county-level. All models were tested using regular Poisson regression and negative binomial regression. The candidate models were compared using cross-validation by year, with out-of-sample log-likelihood as the selection criterion. The selected model was a negative binomial regression specification, containing *a*; *a*^*2*^*; F*, dummies indicating adult (18 years of age; *A*) vs. child (< 18 years of age; *C*) vs. toddler (3 years of age; *T*), and *Y*:

$$ \log (Incidence)=A\left({\alpha}_A+{\beta}_{y,A}Y+{\beta}_{a,A}a+{\beta}_{a^2,A}{a}^2+F\left({\alpha}_{F,A}+{\beta}_{F,Y,A}Y+{\beta}_{F,a,A}a+{\beta}_{F,{a}^2,A}{a}^2\right)\right)+C\left({\alpha}_C+{\beta}_{a,C}a+T\left({\alpha}_T+{\beta}_{a,T}a\right)\right)+\varepsilon $$

This model specification allows for direct comparison and significance testing of differences between men and women. As we were not concerned with comparing stroke rates in children with rates for adults, we used two dummies (*C* and *A*, where *C* = 1-*A*), equivalent to splitting the data into one set for children and one for adults, and running separate models on the two sets. Note that the model for children does not include any terms for time trend. The child model is based on a separate, comprehensive study of strokes in the young, which concludes that there is no observable time trend for this group [22].

We estimated 12 different models: 10 relating exclusively to hospitalizations, i.e. year-unique individuals and hospital episodes for any stroke, I61, I63, I64, and G45; year-unique individuals with any stroke including deaths with no prior hospitalization; and all episodes for any stroke including deaths with no prior hospitalization.

### Predictions and projections

We applied the model for number of year-unique individuals hospitalized with any stroke to population projections from Statistics Norway for 2018–2040. The projections are presented graphically, both with the estimated time trend unchanged, and assuming that the estimated decline in risk will stop at 2015-level.

### Software

All statistical analyses were performed in the R statistical package, version 3.3.2, in the RStudio environment, using ggplot for graphical output [23–25]; negative binomial regression models were fitted with the MASS-package’s glm.nb-function [26].

## Results

Between 2010 and 2015, the population of Norway increased from 4.86 M to 5.17 M (6.4%), and the number of individuals aged 60 and above increased from 0.96 M to 1.06 M (10.4%). The data covers 30.1 M person-years at risk (PY). Between 2010 and 2015, 53,431 unique individuals were admitted to Norwegian hospitals with a primary stroke diagnosis (I61, I63, I64), and 6315 additional individuals were registered as dead due to stroke, for a total of 59,746 unique individuals.

**Fig. 1:**
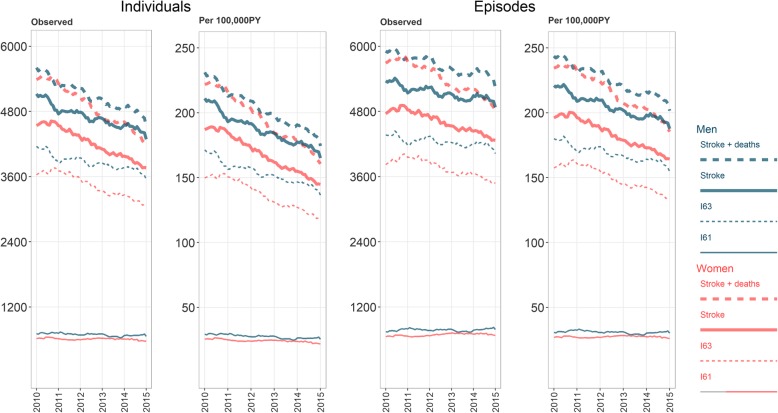
12-month moving sum of observed strokes by subtype, sex, and year. Numbers for men in blue, women in red. Thick solid lines: hospitalizations from any stroke. Thick dotted: Any stroke including deaths. Thin dotted: ischemic strokes. Thin solid: hemorrhagic stroke. Individuals: year-unique individuals. Episodes: All hospitalizations

The number of patients diagnosed with strokes fell substantially between 2010 and 2015, from 11,603 (10,121 excluding immediate deaths) in 2010 to 10,058 (9150 excluding immediate deaths) in 2015 (see Table 1 A and Fig. 1). This corresponds to a drop from 239 to 195 per 100,000 PY (208 to 177 excluding immediate deaths). The number of treatment episodes fell from 13,950 (12,133 excluding immediate deaths) in 2010 to 11,798 (10,645 excluding immediate deaths) in 2015 (see Table 1 B). Adjusted to the WHO and European standard populations, the same picture emerges (Table 2).

**Table 1:** Year-unique individuals and stroke episodes, plus stroke deaths (cases per 100,000 person-years

Year	2010	2011	2012	2013	2014	2015
A) Year-unique individuals						
Men						
Hemorrhage^a^	747 (30.8)	797 (32.4)	766 (30.7)	789 (31.1)	774 (30.1)	786 (30.2)
Infarction^b^	4368 (180)	4179 (169.8)	4340 (173.7)	4228 (166.7)	4219 (164.3)	4024 (154.8)
Unknown^c^	404 (16.6)	320 (13)	256 (10.2)	220 (8.7)	220 (8.6)	175 (6.7)
Any stroke^d^	5364 (221)	5141 (208.9)	5240 (209.7)	5121 (201.9)	5089 (198.2)	4878 (187.7)
+ deaths^e^	5913 (243.7)	5719 (232.4)	5781 (231.3)	5588 (220.4)	5545 (216)	5239 (201.6)
TIA^f^	2249 (92.7)	2273 (92.4)	2207 (88.3)	2328 (91.8)	2217 (86.4)	2207 (84.9)
Women						
Hemorrhage^a^	652 (26.8)	662 (26.9)	671 (27)	721 (28.7)	700 (27.5)	676 (26.3)
Infarction^b^	3822 (157.2)	3954 (160.8)	3876 (155.9)	3677 (146.2)	3658 (143.9)	3479 (135.5)
Unknown^c^	448 (18.4)	384 (15.6)	317 (12.7)	274 (10.9)	210 (8.3)	231 (9)
Any stroke^d^	4757 (195.6)	4828 (196.3)	4699 (188.9)	4536 (180.3)	4438 (174.6)	4272 (166.4)
+ deaths^e^	5690 (234)	5716 (232.4)	5600 (225.2)	5253 (208.8)	5206 (204.8)	4819 (187.7)
TIA^f^	2254 (92.7)	2287 (93)	2418 (97.2)	2366 (94.1)	2229 (87.7)	2098 (81.7)
Total						
Hemorrhage^a^	1399 (28.8)	1459 (29.7)	1437 (28.8)	1510 (29.9)	1474 (28.9)	1462 (28.3)
Infarction^b^	8190 (168.6)	8133 (165.3)	8216 (164.8)	7905 (156.5)	7877 (154.2)	7503 (145.2)
Unknown^c^	852 (17.5)	704 (14.3)	573 (11.5)	494 (9.8)	430 (8.4)	406 (7.9)
Any stroke^d^	10,121 (208.3)	9969 (202.6)	9939 (199.3)	9657 (191.2)	9527 (186.5)	9150 (177.1)
+ deaths^e^	11,603 (238.8)	11,435 (232.4)	11,381 (228.3)	10,841 (214.6)	10,751 (210.4)	10,058 (194.7)
TIA^f^	4503 (92.7)	4560 (92.7)	4625 (92.8)	4694 (92.9)	4446 (87)	4305 (83.3)
B) Yearly stroke episodes separated by at least 24 h						
Men						
Hemorrhage^a^	814 (33.5)	856 (34.8)	845 (33.8)	854 (33.7)	832 (32.4)	898 (34.6)
Infarction^b^	5418 (223.3)	4817 (195.7)	4977 (199.2)	4895 (193)	4944 (192.6)	4874 (187.5)
Unknown^c^	413 (17)	324 (13.2)	277 (11.1)	232 (9.1)	222 (8.6)	190 (7.3)
Any stroke^d^	6521 (268.7)	5879 (238.9)	6017 (240.8)	5891 (232.3)	5909 (230.2)	5882 (226.3)
+ deaths^e^	7195 (296.5)	6602 (268.3)	6692 (267.8)	6490 (255.9)	6496 (253)	6347 (244.2)
TIA^f^	2352 (96.9)	2377 (96.6)	2319 (92.8)	2492 (98.3)	2334 (90.9)	2371 (91.2)
Women						
Hemorrhage^a^	713 (29.3)	724 (29.4)	744 (29.9)	769 (30.6)	776 (30.5)	716 (27.9)
Infarction^b^	4587 (188.7)	4450 (180.9)	4463 (179.5)	4160 (165.4)	4138 (162.8)	3901 (152)
Unknown^c^	453 (18.6)	388 (15.8)	325 (13.1)	276 (11)	213 (8.4)	235 (9.2)
Any stroke^d^	5612 (230.8)	5424 (220.5)	5402 (217.2)	5099 (202.7)	5028 (197.8)	4763 (185.5)
+ deaths^e^	6755 (277.8)	6558 (266.6)	6547 (263.2)	6062 (241)	6020 (236.9)	5451 (212.4)
TIA^f^	2394 (98.5)	2385 (97)	2536 (102)	2463 (97.9)	2334 (91.8)	2228 (86.8)
Total						
Hemorrhage^a^	1527 (31.4)	1580 (32.1)	1589 (31.9)	1623 (32.1)	1608 (31.5)	1614 (31.2)
Infarction^b^	10,005 (205.9)	9267 (188.3)	9440 (189.3)	9055 (179.3)	9082 (177.8)	8775 (169.9)
Unknown^c^	866 (17.8)	712 (14.5)	602 (12.1)	508 (10.1)	435 (8.5)	425 (8.2)
Any stroke^d^	12,133 (249.7)	11,303 (229.7)	11,419 (229)	10,990 (217.6)	10,937 (214.1)	10,645 (206.1)
+ deaths^e^	13,950 (287.1)	13,160 (267.5)	13,239 (265.5)	12,552 (248.5)	12,516 (245)	11,798 (228.4)
TIA^f^	4746 (97.7)	4762 (96.8)	4855 (97.4)	4955 (98.1)	4668 (91.4)	4599 (89)

^a^ICD 10 code I61, ^b^ICD 10 code I63, ^c^ICD 10 code I64, ^d^ICD 10 codes I61, I63, or I64, ^e^Hospital stays + deaths with stroke as primary diagnosis, ^f^Transient Ischemic Attack, ICD 10 code G45

**Table 2:** WHO (European) standard population (size 100,000) rates

Year	2010	2011	2012	2013	2014	2015
A) Year-unique individuals						
Men						
Hemorrhage^a^	19.8 (42.4)	21.0 (44.6)	19.5 (42)	19.6 (42.8)	19.0 (41.3)	18.9 (40.5)
Infarction^b^	113.5 (255.5)	106.0 (238.8)	108.6 (242)	103.5 (230.1)	100.8 (224)	93.5 (210.7)
Unknown^c^	9.9 (25.4)	7.8 (19.2)	6.2 (15.1)	5.1 (13.3)	5.0 (12.7)	3.8 (9.7)
Any stroke^d^	151.8 (313.6)	143.7 (293.4)	143.0 (291.8)	135.5 (279.3)	131.4 (270.8)	121.1 (255)
+ deaths^e^	139.4 (352.3)	131.0 (333.2)	131.3 (327.6)	125.5 (310.5)	121.9 (300.4)	113.8 (277.7)
TIA^f^	60.1 (126.8)	59.1 (126)	56.1 (119.5)	57.9 (123.2)	53.9 (115.4)	52.5 (111.7)
Women						
Hemorrhage^a^	12.3 (27.3)	12.7 (28)	12.9 (28)	13.8 (30.3)	13.1 (28.8)	12.6 (27.4)
Infarction^b^	67.5 (160.4)	69.4 (164.5)	66.9 (160)	63.7 (150.9)	63.1 (148.1)	59.7 (139.4)
Unknown^c^	6.6 (17.8)	5.7 (15.3)	4.7 (12.5)	4.2 (10.8)	3.1 (8.1)	3.6 (9)
Any stroke^d^	94.6 (199.1)	95.3 (201)	91.8 (194)	87.3 (186.6)	85.7 (180)	79.6 (171.3)
+ deaths^e^	84.1 (234.2)	85.3 (233.8)	82.1 (226.8)	79.6 (212.5)	77.5 (207.5)	74.1 (190.3)
TIA^f^	45.7 (98.6)	45.3 (99.3)	47.5 (103.8)	46.3 (100.8)	42.4 (92.8)	39.6 (86.5)
Total						
Hemorrhage^a^	15.8 (34.1)	16.6 (35.5)	16.0 (34.2)	16.5 (35.7)	15.9 (34.4)	15.6 (33.4)
Infarction^b^	88.9 (202.4)	86.7 (198.9)	86.9 (198)	82.8 (188)	81.2 (184)	75.7 (172.2)
Unknown^c^	8.1 (21)	6.7 (17.2)	5.4 (13.7)	4.6 (11.9)	3.9 (10.1)	3.7 (9.3)
Any stroke^d^	121.4 (249.7)	118.1 (243.6)	116.2 (238.9)	110.3 (229.5)	107.6 (222.5)	99.3 (209.9)
+ deaths^e^	109.8 (286.7)	106.9 (279.4)	105.5 (273.6)	101.4 (257.8)	98.7 (251.2)	92.9 (230.7)
TIA^f^	52.4 (111.1)	51.9 (111.5)	51.7 (111.2)	51.8 (111)	47.9 (103.2)	45.8 (97.9)
B) Yearly stroke episodes separated by at least 24 h						
Men						
Hemorrhage^a^	21.4 (46.1)	22.6 (47.9)	21.6 (46.4)	21.4 (45.8)	20.5 (44.2)	21.4 (46)
Infarction^b^	141.9 (317.9)	122.4 (275.2)	124.4 (277.9)	120.3 (264.4)	117.9 (262.8)	113.5 (254.2)
Unknown^c^	10.2 (25.9)	7.9 (19.4)	6.7 (16.4)	5.4 (14.1)	5.0 (12.8)	4.2 (10.7)
Any stroke^d^	185.8 (381.9)	165.9 (335.5)	165.3 (335.8)	157.7 (318.9)	153.6 (314.7)	146.8 (306.4)
+ deaths^e^	170.6 (429.1)	150.0 (385.3)	150.7 (380.7)	145.0 (358.6)	141.4 (352.5)	137.4 (335.8)
TIA^f^	62.8 (132.7)	61.8 (132)	58.7 (125.8)	61.8 (132.5)	56.4 (121.9)	56.0 (120.4)
Women						
Hemorrhage^a^	13.8 (30)	14.3 (30.8)	14.6 (31.1)	14.8 (32.4)	14.7 (32.3)	13.5 (29.1)
Infarction^b^	82.4 (192.8)	77.0 (183.6)	77.0 (184.8)	73.0 (171.1)	72.2 (168)	67.6 (156.8)
Unknown^c^	6.7 (18)	5.8 (15.5)	4.9 (12.8)	4.2 (10.9)	3.1 (8.2)	3.7 (9.2)
Any stroke^d^	114.0 (235.4)	107.7 (224.7)	107.0 (223.6)	100.9 (210.3)	99.3 (204.7)	90.4 (191.7)
+ deaths^e^	101.1 (278.2)	95.2 (266.3)	94.6 (265.3)	90.5 (245.1)	88.7 (240.1)	83.5 (215.5)
TIA^f^	48.1 (104.4)	47.1 (103.4)	49.4 (108.7)	48.0 (104.9)	44.1 (97.1)	42.0 (91.9)
Total						
Hemorrhage^a^	17.3 (37)	18.2 (38.6)	17.8 (37.9)	17.9 (38.3)	17.5 (37.6)	17.3 (36.9)
Infarction^b^	110.2 (248.8)	98.5 (226.1)	99.7 (228)	95.6 (214.8)	93.9 (212.1)	89.4 (201.7)
Unknown^c^	8.3 (21.4)	6.8 (17.4)	5.7 (14.5)	4.7 (12.3)	4.0 (10.2)	3.9 (9.9)
Any stroke^d^	147.5 (300.7)	135.2 (275.9)	134.8 (275.2)	128.0 (260.7)	125.1 (255.5)	117.1 (244.6)
+ deaths^e^	133.5 (345.9)	121.1 (321.1)	121.3 (319)	116.5 (297.9)	113.7 (292.5)	109.0 (271.1)
TIA^f^	55.1 (117.1)	54.1 (116.5)	53.9 (116.7)	54.5 (117.4)	50.0 (108.3)	48.7 (104.7)

^a^ICD 10 code I61, ^b^ICD 10 code I63, ^c^ICD 10 code I64, ^d^ICD 10 codes I61, I63, or I64, ^e^Hospital stays + deaths with stroke as primary diagnosis, ^f^Transient Ischemic Attack, ICD 10 code G45

The inclusion of patients from the NCDR records contributed approximately 1000 unique individuals afflicted by stroke per year. In hospitalized patients, the largest relative decline was for unspecified stroke, which was approximately halved over the 6-year period. The number of individuals hospitalized with ischemic stroke fell by 7.9% for men and 9.0% for women over the 6-year period. The number of hemorrhagic stroke and TIAs were relatively unchanged.

The estimated models for stroke incidence can be found in Table 3. The models indicate a near-exponential increase in stroke risk by age in the adult population, with relatively higher risk for men than women. The incidence of ischemic strokes and any stroke display a marked decline over time, with the difference in time trend between men and women statistically non-significant. For hemorrhagic stroke, the time trend was non-significant for both men and women.

**Table 3:** Negative binomial regression models predicting yearly risk of strokes (+ TIA) by type

	Hemorrhage^d^	Infarction^e^	Unknown^f^	TIA^g^	Any stroke^c^	Any incl. Death
Individuals^a^						
Adult (age > 17)						
Constant	−13.517***	−14.4230***	−16.2628***	−15.2673***	−13.6616***	−13.1802***
Year since 2010	−0.016	− 0.0346***	− 0.1839***	− 0.0262***	− 0.0379***	− 0.0437***
Age (years)	0.112***	0.1879***	0.1415***	0.2061***	0.1710***	0.1534***
Age^b^	0.000***	−0.0008***	− 0.0003*	− 0.0010***	− 0.0006***	− 0.0005***
Female	−0.842*	0.5912**	0.2203	−0.4282	0.3496*	0.5552***
Female * year	0.016	0.0030	0.0223	0.0016	0.0043	0.0005
Female * age	0.009	− 0.0432***	− 0.0231	0.0009	− 0.0340***	−0.0414***
Female * age^b^	0.000	0.0004***	0.0002	0.0000	0.0003***	0.0004***
Child (age < 18)						
Constant	−12.401***	−12.0622***	−13.2741***	−14.3341***	−11.4178***	−11.3493***
Toddler (age < 4)	2.436***	2.9728***	−0.3955	1.3481	2.6754***	2.6069***
Age	0.068	0.0405	−0.0818	0.1682**	0.0467	0.0425
Age * toddler	−0.984***	−1.1838***	−0.3499	0.2416	−1.1106***	−1.1063***
Episodes^b^						
Adult (age > 17)						
Constant	−13.636***	−14.3582***	−16.5349***	−15.2870***	−13.6388***	−13.1495***
Year since 2010	−0.009	−0.0370***	−0.1768***	−0.0232***	−0.0384***	−0.04353***
Age (years)	0.119***	0.1921***	0.1504***	0.2068***	0.1762***	0.15748***
Age^b^	0.000***	−0.0008***	−0.0003**	−0.0010***	−0.0007***	−0.00051***
Female	−0.506	0.8220***	0.4655	−0.3954	0.6176***	0.7991***
Female * year	0.006	0.0018	0.0176	−0.0012	0.0024	−0.00061
Female * age	0.001	−0.0491***	−0.0304	0.0004	−0.0409***	−0.04815***
Female * age^b^	0.000	0.0004***	0.0003	0.0000	0.0003***	0.00041***
Child (age < 18)						
Constant	−12.248***	−11.6127***	−13.1404***	−14.3342***	−11.0972***	−11.0428***
Toddler (age < 4)	2.505***	2.5881***	−0.5291	1.3484	2.4693***	2.41516***
Age	0.061	0.0220	−0.0722	0.1682**	0.0327	0.02939
Age * toddler	−0.976***	−1.0107***	−0.3597	0.2414	−0.9958***	−0.99262***

Statistical significance: ““> 0.05 > = “*” > 0.01 > = “**” > 0.001 > = “***”

^a^Year-unique individuals, ^b^Recorded hospitalizations or deaths separated by at least 24 h,^c^ICD-10 codes I61, I63, or I64, ^d^ ICD-10 code I61, ^e^ ICD-10 code I63, ^f^ ICD-10 code I64, ^g^ Transient Ischemic Attack - ICD-10 code G45

**Fig. 2:**
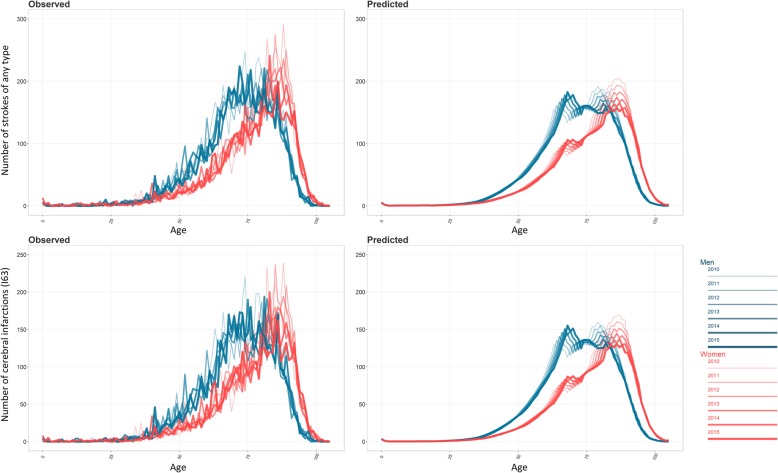
Observed and predicted number of strokes by age, sex, and year

Observed and predicted number of individuals hospitalized with any stroke and ischemic stroke as a function of sex, age, and year are juxtaposed in Fig. 2.

**Fig. 3:**
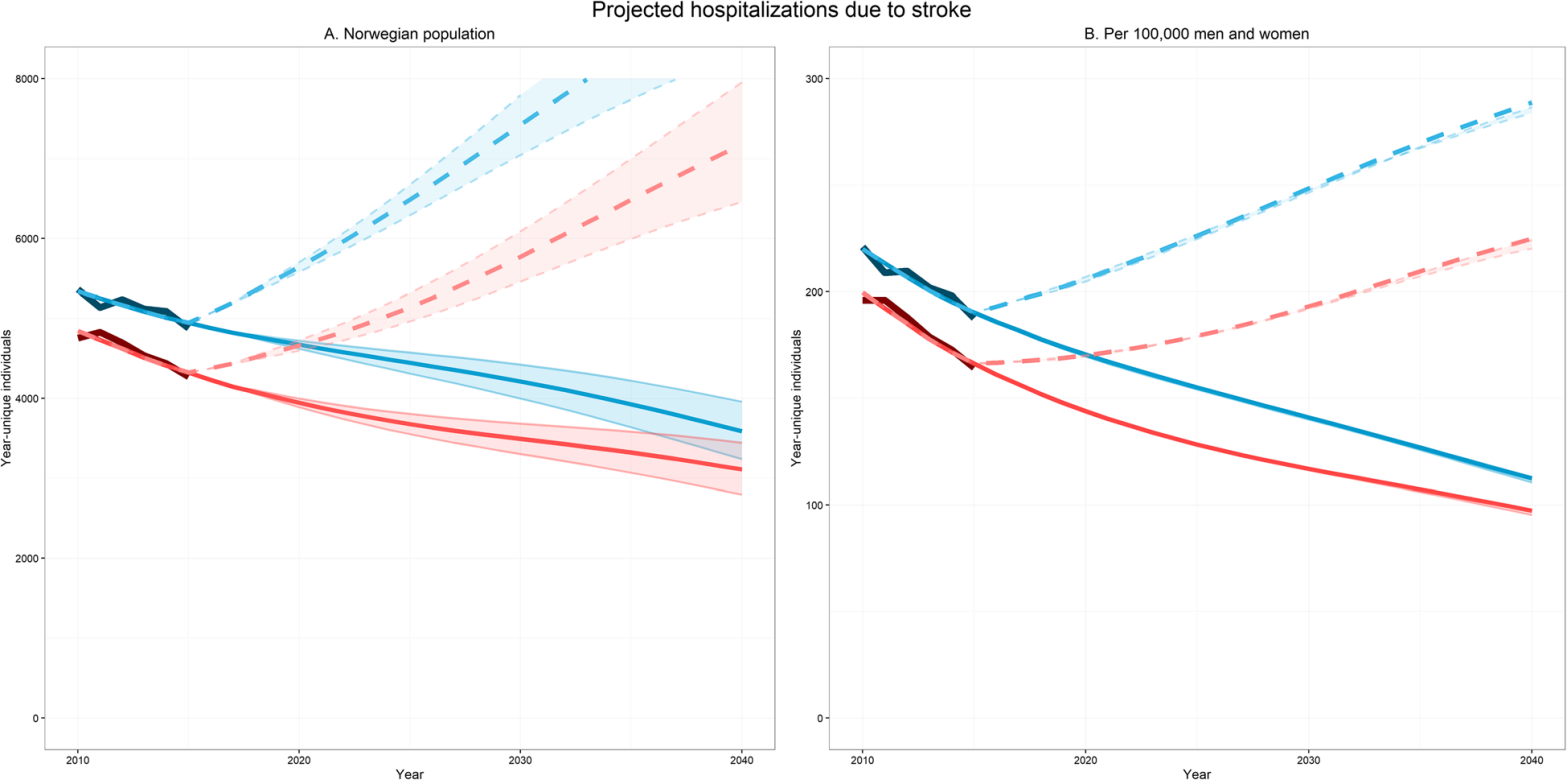
Projected number of year-unique individuals hospitalized due to stroke 2010–2040. Blue: men. Red: women. Dark lines: observed. Thick solid: predictions based on Statistics Norway’s main population projections and fitted regression model in Table 3, column labelled “Any stroke”. Dashed: time trend in model fixed at 2015-level; changes due to projected ageing and population growth only. Colored bands are between Statistics Norway’s population projections for high and low population growth

Figure 3 displays projections for total number of men and women hospitalized due to stroke 2018–2040 based on applying the fitted model to the main population projections from Statistics Norway. Projecting the fitted time trend into the future indicates a continued drop in overall stroke incidence. If the decline in risk observed between 2010 and 2015 does not continue, the number of individuals suffering stroke will increase substantially due to ageing of the population.

Projections and fitted values based on all 12 fitted models for 2010–2040 are provided in an online .csv file (Additional file 1).

## Discussion

Between 2010 and 2015, the incidence of hemorrhagic stroke remained relatively unchanged, while ischemic stroke incidence decreased substantially. At present, the reduction in the rates for ischemic stroke seems sufficient to out-pace the growth and ageing of the population. Extrapolating the fitted incidence models backwards towards 2000 using the recorded population at the time suggests that there should have been around 18,300 strokes in Norway at the time. This overshoots contemporary incidence estimates for Norway of around 15,000 yearly cases [10] by a substantial margin, suggesting that the rate of decline may have accelerated. If the observed trend continues unabated, there will be a continued decline in the overall stroke incidence in Norway over the next decades. However, as illustrated by Fig. 3, this depends crucially on the underlying risk of ischemic stroke to continue its decline; the population is projected to age substantially over the next decades, meaning that the number of individuals in high-risk age groups will increase dramatically. If the decline in ischemic stroke incidence were to level out, the number of stroke cases should be expected to increase at an accelerating rate. Studies on time trends in stroke incidence from the last decades have shown a decreasing trend in incidence in high-income countries, and the present study confirms that this trend has continued since 2000. Declining incidence of ischemic stroke may be attributed to various factors, such as improved primary and secondary prevention, including earlier detection and treatment of atrial fibrillation. In previous studies, the decline attributed to specific risk factors ranges from 55% in a population based study [27] to 82% in large case-control [28]. The authors of the Tromsø study from Norway reported a decrease of nearly 20% in stroke incidence from 1995 to 2012, and estimated that reduction of blood pressure and reduced prevalence of smoking accounted for 43% of the observed decline [29]. In this study daily smoking dropped with 34% in one decade. Increasing prevalence of high BMI and diabetes were found to increase stroke incidence, but their impact was insufficient to outweigh the benefit of other risk reductions.

As previously noted, the incidence and risk of hemorrhagic stroke was relatively stable over the observed 6-year period. Where great improvements have been made in terms of reducing or ameliorating risk factors for ischemic stroke, it appears that risk factors for hemorrhage have seen smaller improvements. We should point out, however, that the he use of the undefined stroke type as diagnostic code for live patients has been close to eradicated. Presumably, most patients that would previously have been diagnosed as ‘undetermined’ now reside in the ischemic- or hemorrhagic stroke categories. Consequently, if hemorrhagic stroke incidence was fixed, we should have expected an observed increase in incidence from patients that would previously have been diagnosed with stroke of undetermined type. Changes in diagnostic accuracy during the study period is a potential concern for estimates of change in incidence. Unlike hemorrhagic strokes, that are immediately visible on imaging, ischemic stroke diagnoses may still be based on clinical evaluation combined with a normal CT scan. With increasing use of MRI and multiple images, the ability to rule out ischemia has improved. Consequently, a subgroup of patients that were previously diagnosed with an ischemic stroke based on clinical evaluation and CT scans may now be correctly characterized as stroke mimics [30]. The observation period falls within a period of increased attention to strokes and stroke treatment in Norway, as reflected by the decision to make the Norwegian stroke registry, which was established in 2004, a compulsory national registry in 2012 [31], and various efforts for increasing stroke symptom awareness, and knowledge about the time is brain paradigm [32]. Increased awareness and attention may have contributed to changes both in diagnostic practice and coding, potentially contributing to the observed time trends. Similarly, we cannot rule out potential changes in the accuracy of death certificates over the observed time period. However, we are unaware of any specific factors occurring within the observation period that would suggest substantial changes. The continued decline in stroke incidence observed in this study is most likely attributable to changes in underlying risk factors over the last decades.

An alternative hypothesis for the observed decline in the incidence of ischemic stroke would be that a subset of patients previously identified as ischemic strokes would now be diagnosed as having had a TIA. This hypothesis does not correspond well with perceptions among clinicians, or with the adaptation of more sensitive imaging equipment, both of which would be expected to produce a shift in the opposite direction. As reflected in the tables and the regression models for TIA, there was a downward trend in both the number of hospitalizations and year-unique individuals admitted due to TIA between 2010 and 2015, supporting the notion that the declining incidence of ischemic stroke is not caused by a shift in the delineation between ICD-10 codes G45 (TIA) and I63 (ischemic strokes). One potential concern for our observations of trends for TIA is the exclusion of outpatient visits from our analyses. While there may be instances in which patients experiencing a TIA were to visit the hospital as an outpatient only, this would require breach of national guidelines: suspected cerebral incidents are to be admitted. With increasing awareness and better diagnostic methods, the threshold for admission to hospital with suspected TIA has likely been lowered over the time frame in question. Since the diagnostic codes used do not specify if the stroke in question is the first suffered by the patient, and our observational period is limited to 6 years, we cannot directly ascertain time trends in the incidence or proportion of first ever strokes.

## Conclusion

In conclusion, the declining trend observed in the incidence of ischemic strokes in high income countries has continued in Norway, at least up until 2015. Between 2010 and 2015, the rate of decline outpaced the growth and ageing of the population.

## Supplementary information

**Additional file 1:** Incidence_predictions.csv. Predictions for strokes and TIA in Norway by type, (I61, I63, I64, G45) year (2010–2040),and age (0–105). The data contains the following columns: Year: Years from 2010 to 2040. Age: Age from 0 to 105. obs_population: observed size of Norwegian population of specified age by year, for 2010–2017. Pred_population: predicted population size for specified age for years 2018–2040. Twelve columns of predictions, identified by the following: DIAGNOSIS_pred_TYPE. DIAGNOSIS includes I61, I63, I64, G45 and “stroke” (any of I61, I63, I64). TYPE indicates as “episodes” (yearly hospitalizations) and “year_unique” (unique individuals afflicted that year). For diagnosis “stroke”, there are additional columns including “with_death”, signifying episodes or year-unique individuals including those dying with no preceding hospitalization. Final 12 columns specify the estimated individual yearly risk of the predicted stroke types.

## Abbreviations

ACI: Acute Cerebral Infarction
BMI: Body Mass Index
CT: Computer Tomography
CVA: Cerebrovascular Accident
ICD-10: International Classification of Diseases, version 10
ICH: Intracerebral Hemorrhage
MRI: Magnetic Resonance Imaging
NCDR: Norwegian Cause of Death Registry
NIPH: (Norwegian) National Institute of Public Health
NPR: Norwegian Patient Registry
PY: Person-years
TIA: Transient Ischemic Attack
WHO: World Health Organization
